# Interleukin 6 for the Prediction of Chorioamnionitis: A Systematic Review and Meta-Analysis

**DOI:** 10.3390/biomedicines13112577

**Published:** 2025-10-22

**Authors:** Eleni Solomou, Emmanouil Kalampokas, Christos Michailides, Theodoros N. Sergentanis, Theodoros Kalampokas

**Affiliations:** 1 Department of Obstetrics and Gynecology, Panarkadikon General Hospital, 221 00 Tripoli, Greece; 2 Unit of Gynecologic Oncology, Second Department of Obstetrics and Gynecology, Aretaieion Hospital, 115 28 Athens, Greece; 3 Department of Internal Medicine, University Hospital of Patras, 265 04 Ρiο, Greece; 4 Department of Hygiene, Epidemiology, and Medical Statistics, School of Medicine, University of Athens, 106 79 Athens, Greece; 5 Unit of Obstetrics and Gynecology, Second Department of Obstetric s and Gynecology, Aretaieion Hospital, 115 28 Athens, Greece

**Keywords:** interleukins, IL-6, prediction, diagnostic accuracy, chorioamnionitis, HCA

## Abstract

**Background:** Chorioamnionitis is the inflammation of the placenta, amniotic fluid, and fetal membranes and its histological confirmation, histologic chorioamnionitis (HCA) is defined as the diffuse infiltration of neutrophils into the chorioamniotic membranes. Several biomarkers have been evaluated for its early prediction, including interleukin-6 (IL-6), which can be measured in plasma, amniotic fluid, and cervicovaginal fluid (CVF). **Aims and Scope:** We aimed to systematically review and meta-analyze the role of IL-6 in the prediction of HCA, in several body fluids and among distinct subgroups. **Methods:** A literature search was conducted in PubMed, Embase, Cochrane Library, and CT.gov between March 2024 and July 2024. Studies that measured IL-6 in AF, CVF, or plasma and conducted a placental examination were included. The Quality Assessment of Diagnostic Accuracy Studies—2 (QUADAS-2) tool was used to assess methodological quality. Bivariate analysis combined with a linear mixed model was used for quantitative synthesis, and summary estimates were calculated. Summary Receiver Operating Characteristic (SROC) curves were constructed to evaluate diagnostic accuracy. The z-test was used for subgroup comparisons. **Results:** In total, 43 studies were included in this meta-analysis, 23 for amniotic fluid (AF), 9 for plasma, and 11 for CVF. AF IL-6 in the overall population had a very good diagnostic performance with an AUC = 0.82 (95% CI: 0.78–0.85) for HCA prediction, with a sensitivity of 65% (95% CI: 0.55–0.74) and a specificity of 84% (95% CI: 0.76–0.89), performing superiorly for the preterm labor (PTL) group (Area Under Curve (AUC) = 0.88, 95% confidence interval (CI): 0.85–0.91) compared with the Preterm premature rupture of membranes (PPROM) subgroup (AUC = 0.76, 95% CI: 0.72–0.80). Plasma IL-6 in the overall population had a good diagnostic performance with an AUC = 0.79 (95% CI: 0.76–0.83), similar to that for the PTL and PPROM subgroups, with a sensitivity of 72% (95% CI: 0.58–0.83) and a specificity of 79% (95% CI: 0.72–0.84). CVF IL-6 in the PPROM group had an excellent diagnostic accuracy, the highest observed in our research (AUC = 0.91, 95% CI: 0.88–0.93), higher than CVF in the overall population, where diagnostic accuracy remained very good. The QUADAS-2 tool revealed a high risk of bias overall. **Conclusions:** CVF IL-6 could serve as a valid, non-invasive screening test for pregnant women to stratify risk for HCA, while a combination of AF, CVF, and plasma IL-6 could be a tractable diagnostic tool for clinicians, but large-scale Randomized Control Trials are needed to validate this hypothesis.

## 1. Introduction

Chorioamnionitis is the infection that affects the fetal membranes, the amniotic fluid, and the placenta and usually occurs due to a premature rupture of the membrane; however, that is not a necessary occurrence for diagnosis. It is often multimicrobial and complicates a significant number of births [1]. In 1982, Gibbs et al. suggested a set of clinical criteria for chorioamnionitis, including maternal fever and leukocytosis, maternal or fetal tachycardia, uterine tenderness, and purulent vaginal discharge combined with a positive amniotic fluid Gram stain or positive culture [2,3]. However, those criteria are nonspecific, and their low diagnostic accuracy raises significant limitations regarding prompt diagnosis and treatment to avoid complications. HCA has been used to define the diffuse infiltration of neutrophils into the chorioamniotic membranes. The histological analysis of the placenta is regarded as the gold standard for diagnosing such kinds of infections, but this result can only be obtained postpartum [4].

Several biomarkers have been tested for their ability to contribute to the diagnosis of chorioamnionitis. Lactate dehydrogenase (LDH) was one of the first predictors tested for the diagnosis of amniotic fluid infection [5]. C-reactive protein (CRP) and Neutrophil-to-Lymphocyte Ratio (NLR) have also been demonstrated as possible diagnostic markers for chorioamnionitis with acceptable diagnostic accuracy that could augment the other diagnostic tools [6]. Interestingly, procalcitonin (PCT) also has adequate sensitivity and specificity to predict HCA among women with preterm premature rupture of membranes (PPROM) [7]. Amniotic fluid and plasma proteins of the matrix metalloproteinase (MMP) family, along with amniotic fluid glucose, have also been studied for their potential to amplify laboratory diagnostic tools with promising results [8,9]. However, there is no recent systematic review that summarizes their diagnostic accuracy, and their specific cut-offs have not been established.

IL-6 is a biomarker that has been tested for numerous conditions and clinical settings. Historically, it is considered a four-helix pro-inflammatory acute-phase cytokine produced by innate immunity cells, which activates host immunity response. IL-6 is involved in chronic and acute inflammation via several signaling pathways, including MAPK, TLR, and JAK, which explains its varied clinical and laboratory significance [10].

IL-6 levels can be detected in pregnancy in several components, including plasma, AF, and CVF and plenty of conditions, such as PPROM and PTL. Consequently, it has been thoroughly studied as a biomarker for further maternal complications, including HCA and microbial invasion of the amniotic cavity (MIAC), and newborn complications, including Fetal Inflammatory Response Syndrome (FIRS) and developmental deformities [11,12,13,14,15]. In this systematic review and meta-analysis, we aimed to investigate the role of IL-6, measured either in the plasma of pregnant women, in amniotic fluid, or in CVF, in the diagnosis of HCA.

## 2. Materials and Methods

This systematic review and meta-analysis used methodological approaches suggested in the *Cochrane Handbook for Systematic Review of Diagnostic Test Accuracy*, followed a prospectively prepared protocol, and adhered to the Preferred Reporting Items for Systematic Reviews and Meta-Analyses (PRISMA) guidelines [16]. We registered our protocol at INPLASY; registration number INPLASY202590110. Comprehensive searches were conducted in the PubMed, Cochrane, CT.gov, and SCOPUS databases up to July 2024, using the following combination of keywords: “((chorioamnionitis) OR (amnionitis) OR (intraamniotic infection) OR (intraamniotic inflammation) OR (HCA) OR (IAI) OR (intrauterine infection)) AND ((IL-6) OR (interleukin-6)) AND ((diagnosis) OR (diagnostic accuracy) OR (Sensitivity) OR (Specificity) OR (prediction) OR (accuracy) OR (PPV) OR (NPV) OR (cut-off) OR (prognosis))”. No language or other restrictions were imposed in the initial search, and no date limits or filters were applied. The gray literature, conference abstracts, and unpublished studies were not included, as standardized reporting of diagnostic accuracy metrics and reference standards was often lacking. Their exclusion was deemed necessary to maintain methodological consistency and ensure the reliability of the synthesized data.

Only studies involving preterm pregnant women that assessed IL-6 in either compartment and conducted a histological evaluation of the placenta post-delivery were deemed eligible. Studies were excluded from further evaluation if they were case reports or case series; however, any study design that compared the results of the index test with the reference standard and provided data suitable for the extraction of 2 × 2 data qualified for inclusion. Additionally, reference lists of pertinent studies were manually searched to identify further relevant studies.

Two independent reviewers (ES and CM) conducted the study selection, and any disagreements regarding eligibility were resolved through consensus discussion with a third reviewer (EK). In each eligible study, the true positives, false negatives, true negatives, and false positives were independently extracted by the two investigators who conducted the literature search (ES and CM). Authors of studies that met the eligibility criteria but contained missing, ambiguous, or conflicting contingency table data were contacted via email for clarification (CM). The search strategy is summarized in Supplementary File S1.

The QUADAS-2 tool was used to assess the risk of bias of each study that reported both sensitivity and specificity metrics [17]. Two reviewers (ES and CM) conducted independent evaluations of QUADAS-2 items, with all identified conflicts resolved through consensus. Meta-analysis was conducted if the number of studies in each sample type category was ≥3. Subsequently, we opted to further analyze patients with PPROM and PTL, respectively, as separate sub-cohorts in order to reduce the likelihood of merging test accuracy indices that might differ due to variations in patient characteristics and probability of disease.

Heterogeneity was assessed visually for studies using the same cut-off by inspecting the 95% prediction regions on SROC curves. Further exploration of heterogeneity was undertaken within each clinical subgroup (PPROM or PTL) when ≥5 studies were available and ≥2 contributed to each category. Potential sources of heterogeneity that we aimed to investigate were optimal cut-offs, time to delivery, and methodological quality (high risk of bias).

A Deeks’ funnel plot or equivalent test for publication bias was not performed, as fewer than ten studies were available for each biological specimen category included in the meta-analysis, limiting the interpretability and statistical power of such assessments.

### Data Synthesis and Statistical Analysis

The pooled sensitivity, specificity, diagnostic odds ratio (DOR), positive (LR+) and negative (LR−) likelihood ratios, as well as the area under the SROC, were estimated for IL-6 using the reported frequencies of true-positive (TP), false-positive (FP), true-negative (TN), and false-negative (FN) results across individual studies. Histological evaluation served as the gold standard for comparison, and 95% confidence intervals (CIs) were calculated for each metric. The estimation was based on a bivariate meta-analysis of sensitivity and specificity utilizing a generalized linear mixed-model approach. Afterward, the DOR and the area under the sROC values were compared between the respective PPROM and PTL subgroups using the appropriate z-tests; 0.05 was chosen as the threshold for statistical significance. STATA/SE version 16 (Stata Corp, College Station, TX, USA) was used for all statistical analyses.

## 3. Results

### 3.1. Study Selection and Study Characteristics

**Figure 1:**
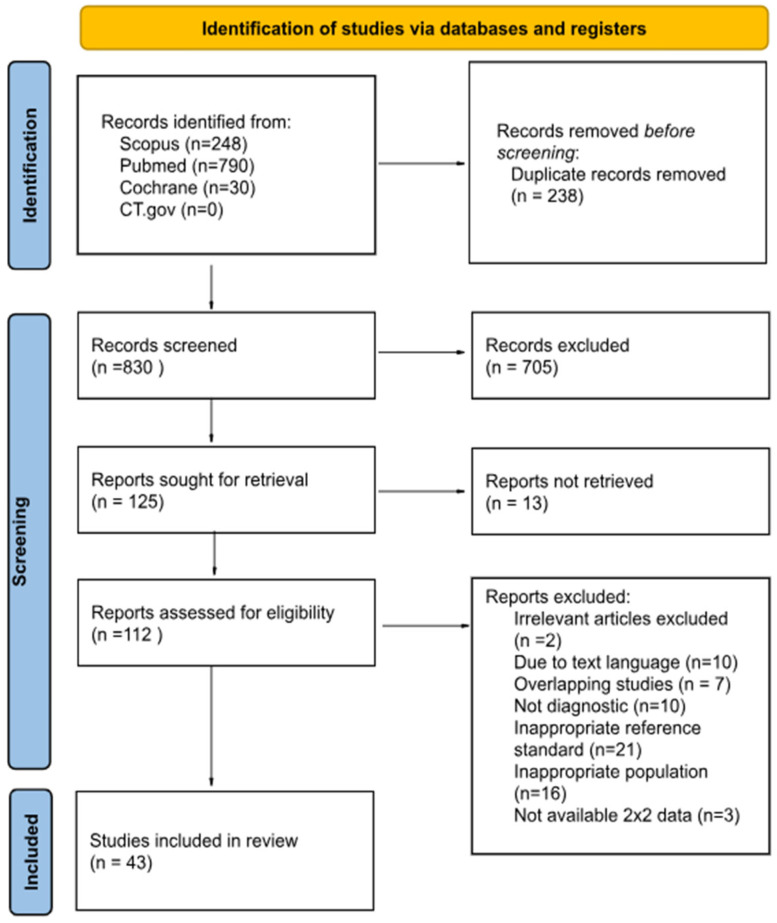
PRISMA flowchart.

Through our search, we identified 1068 respective articles in PubMed, Scopus, and Cochrane Library, of which 830 remained after de-duplication. After the two-step screening process, a total of 43 studies were included [11,12,13,18,19,20,21,22,23,24,25,26,27,28,29,30,31,32,33,34,35,36,37,38,39,40,41,42,43,44,45,46,47,48,49,50,51,52,53,54,55,56,57] (Figure 1). Twenty-three of them were referring to amniotic fluid [11,18,19,20,21,22,23,24,25,26,27,28,29,30,31,32,33,34,35,36,37,38,39]; nine of them examined cervicovaginal fluid [12,40,41,42,43,44,45,46,47], and the remaining eleven analyzed plasma IL-6 [13,48,49,50,51,52,53,54,55,56,57] (Table 1). Overall, 705 articles were excluded due to irrelevance to our study subject during the screening process. Thirteen articles could not be retrieved despite contacting the corresponding authors by email. Ten articles were excluded due to text language. Furthermore, 59 articles were excluded because they were not diagnostic or due to inappropriate populations or reference standards, irrelevance to the study subject, and unavailability of quantitative data despite direct requests from authors.

### 3.2. Quantitative Analysis

**Figure 2:**
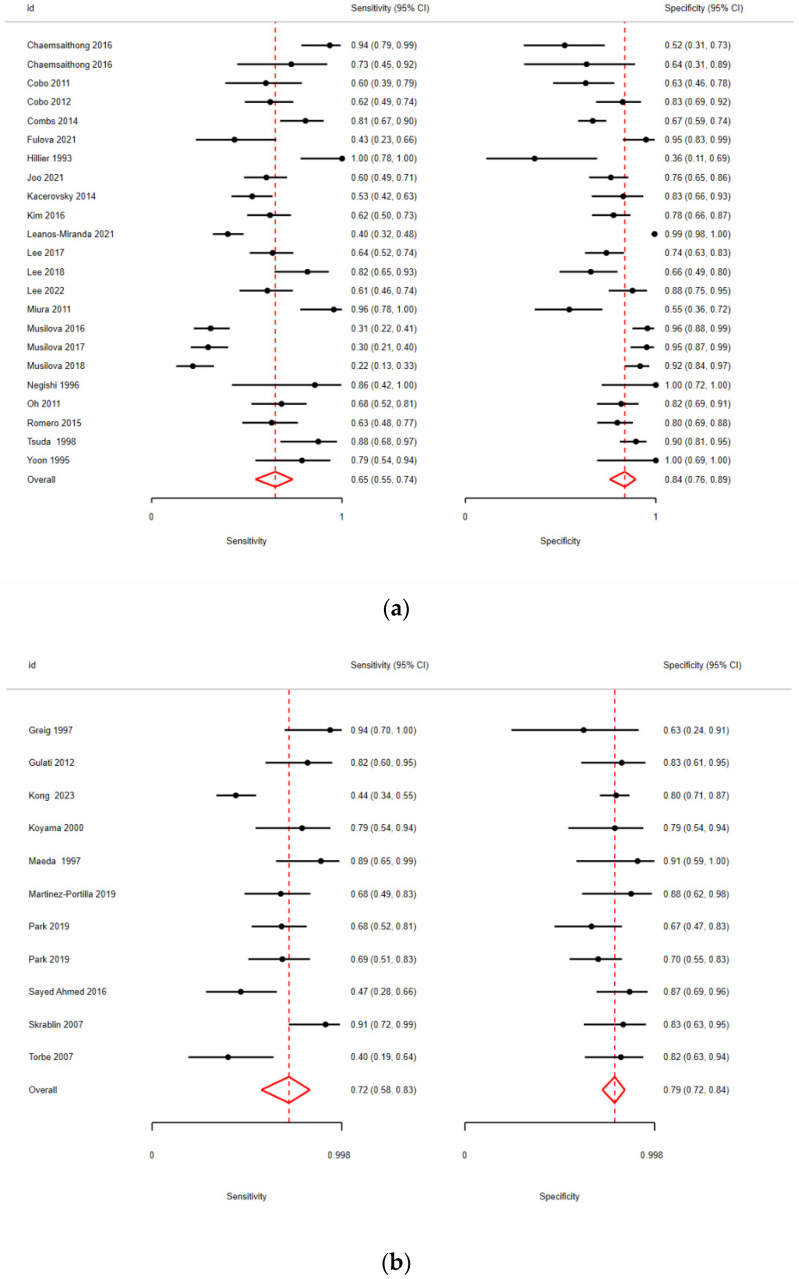

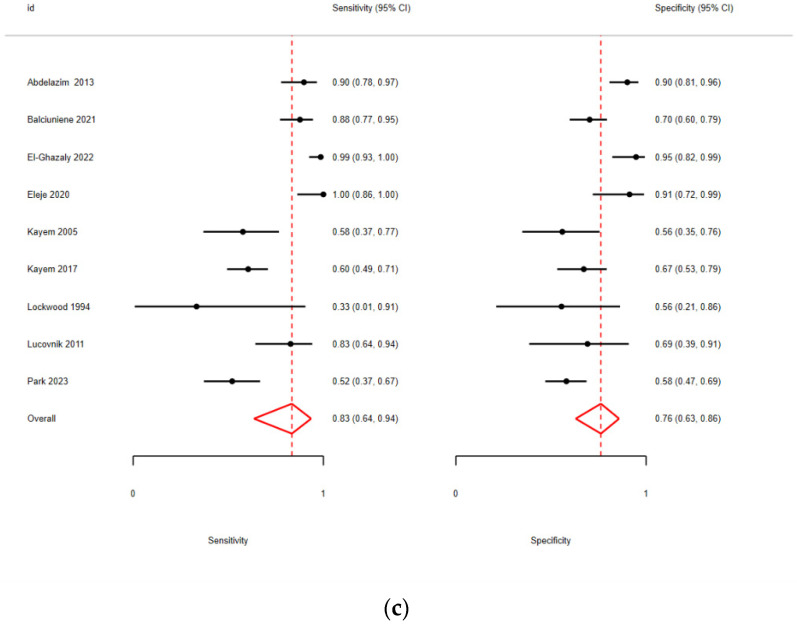
(**a**)**.** Pooled sensitivity and specificity for amniotic fluid (AF) [11,18,19,20,21,22,23,24,25,26,27,28,29,30,31,32,33,34,35,36,37,38,39]; (**b**) pooled sensitivity and specificity for plasma [13,48,49,50,51,52,53,54,55,56,57]; (**c**) pooled sensitivity and specificity for cervicovaginal fluid (CVF) [12,40,41,42,43,44,45,46,47]. Abbreviations: CI: confidence interval.

The pooled sensitivity and specificity for IL-6 in AF were 65% (95% CI: 55–74%) and 84% (95% CI: 76–89%), respectively (Figure 2a), with an AUC of 0.82 (Table 2).

In plasma, the corresponding accuracy parameters were 72% (95% CI: 58–83%) and 79% (95% CI: 72–84%), respectively (Figure 2b), with an AUC = 0.79 (Table 3), while in CVF, they were 83% (95% CI: 64–94%) and 76% (95% CI: 63–86%) (Figure 2c), with an AUC of 0.85 (Table 4).

**Figure 3:**
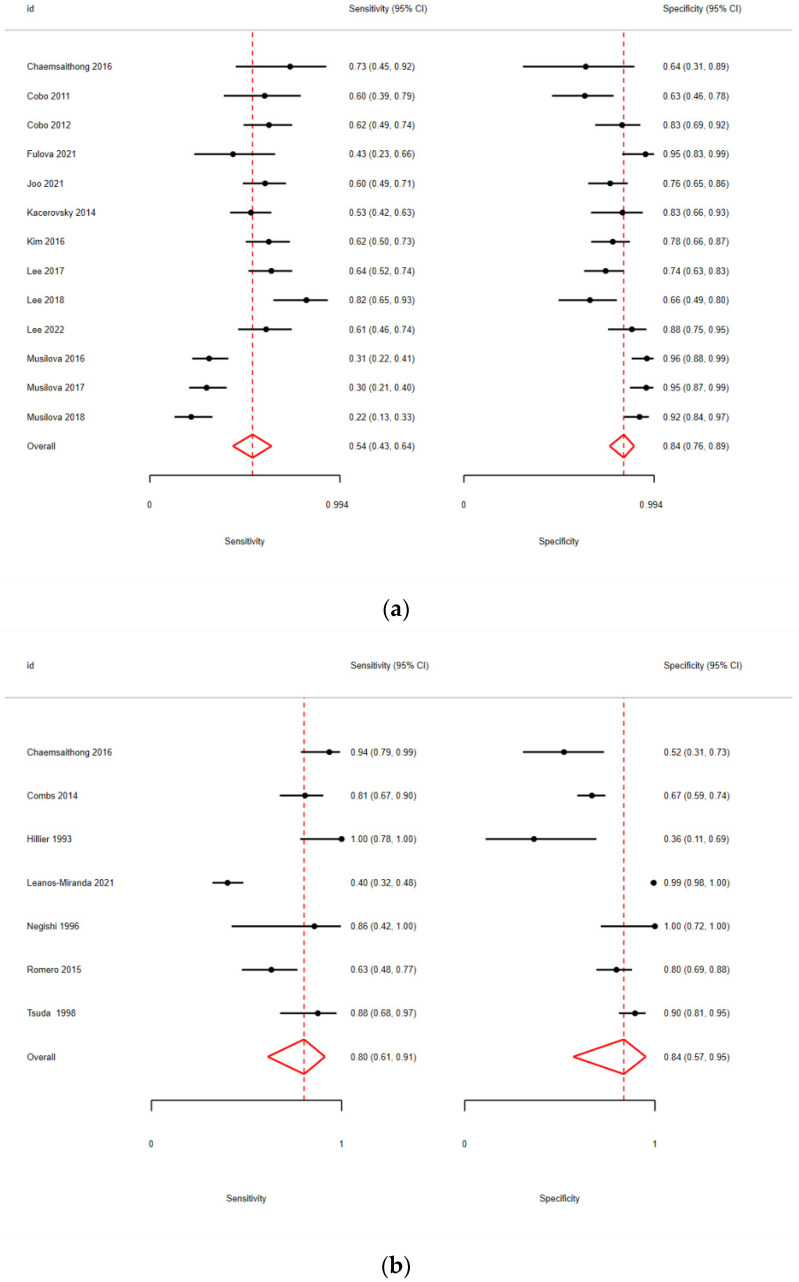
(**a**) Pooled sensitivity and specificity for AF IL-6 in PPROM [11,18,19,20,21,22,23,24,25,26,27,28,29]; (**b**) pooled sensitivity and specificity for AF IL-6 in PTL [30,31,32,33,34,35,36].

Afterwards, AF studies were divided based on their population between PPROM or PTL pregnant women. In PPROM patients, the sensitivity was 54% (95% CI: 43–64%) and the specificity was 84% (95% CI: 76–89%) (Figure 3a), with an AUC = 0.76 (Table 5). In PTL patients, the sensitivity was higher, reaching 80% (95% CI: 61–91%), while the specificity remained consistent at 84% (95% CI: 57–95%) (Figure 3b), with an AUC = 0.88 (Table 5).

A similar approach was taken in plasma studies, but the results did not differ significantly between the subgroups (Table 6).

**Figure 4:**
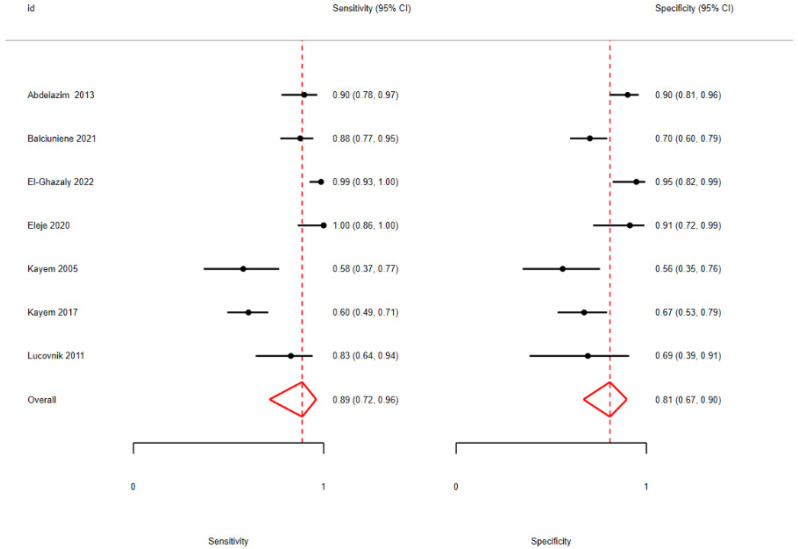
Pooled sensitivity and specificity analysis in CVF PPROM subgroup [12,40,41,42,43,44,45].

The comparison between PPROM and PTL patients with regard to the cervicovaginal fluid (CVF) could not be fully implemented due to a lack of evidence regarding the CVF PTL group, which consisted of fewer than two studies. However, after focusing exclusively on PPROM patients, the diagnostic accuracy metrics demonstrated a significant improvement, indicating an excellent performance for this sub-population with AUC = 0.91 (Table 4, Figure 4).

The model-fitting and heterogeneity statistics for the bivariate meta-analysis model of sensitivity and specificity are summarized in Supplementary Table S1.

**Figure 5:**
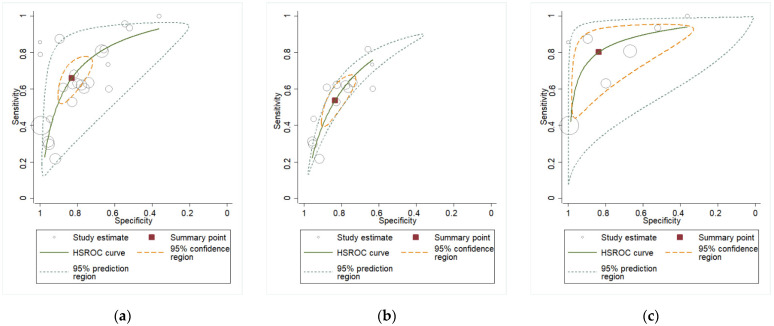
hsROC analysis of IL-6 in AF for (**a**) overall population, (**b**) PTL subgroup, (**c**) PPROM subgroup.

**Figure 6:**
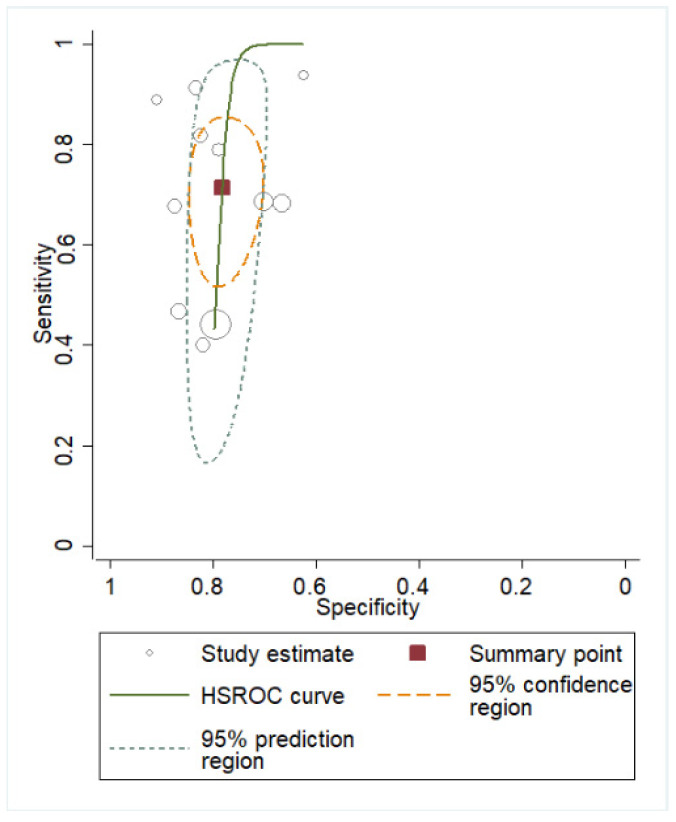
Hierarchical summary receiver operating characteristic (hsROC) analysis of IL-6 in plasma for the overall population.

**Figure 7:**
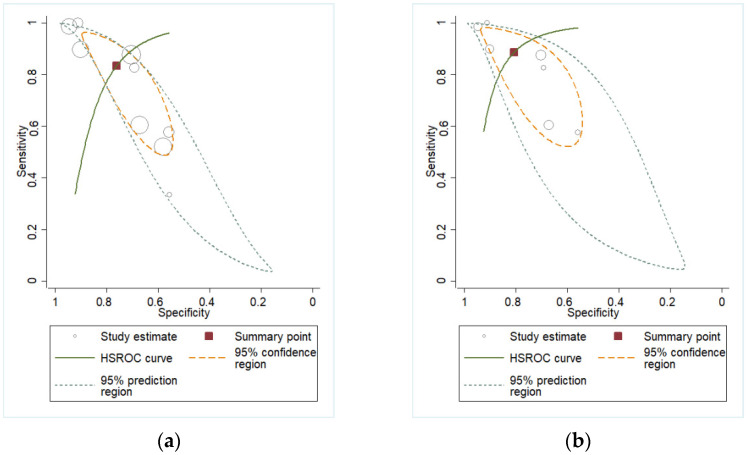
hsROC analysis of IL-6 in CVF for (**a**) overall population, (**b**) PPROM.

Figure 5, Figure 6 and Figure 7 show sROC analyses of IL-6 to predict HCA in the overall AF population, as well as in the AF subgroups of PTL and PPROM, the overall plasma population, and the CVF overall population and PPROM subgroup.

### 3.3. Quality Assessment

**Figure 8:**
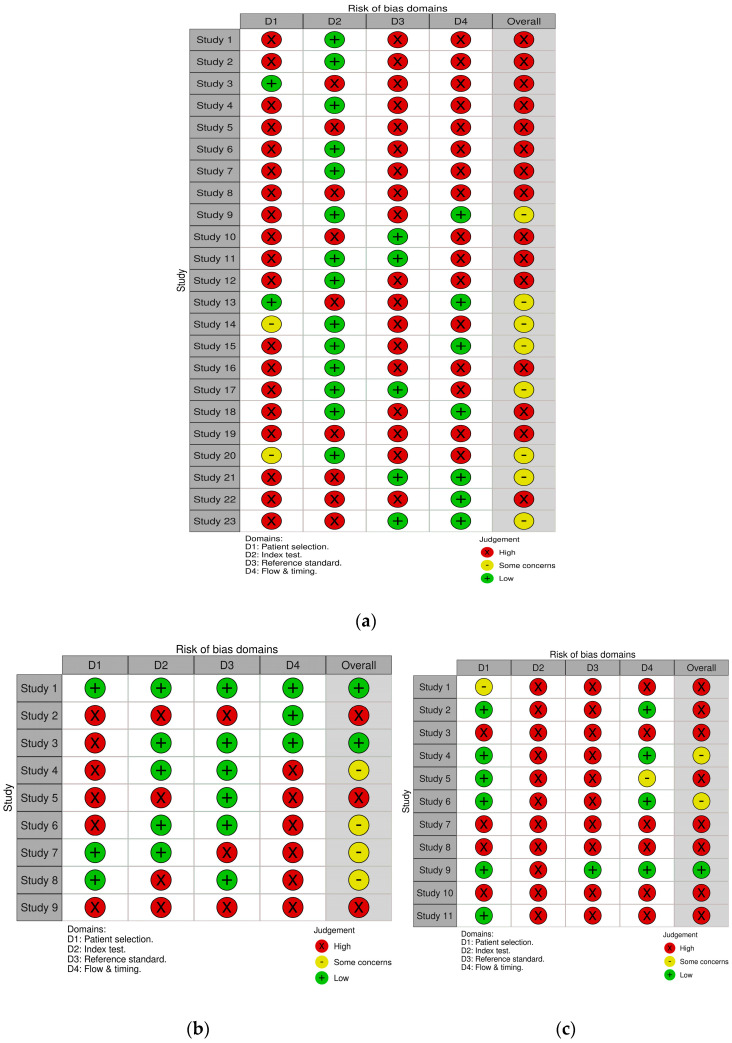
Risk–of–bias concerns graph regarding (**a**) AF, (**b**) CVF, (**c**) plasma.

The quality assessment of the included studies was evaluated using the QUADAS-2 tool, with detailed findings illustrated in Figure 8.

In the “Patient Selection” domain, 29 out of 43 studies were identified as having a high risk of bias [11,12,13,18,20,21,22,23,24,25,26,27,28,29,30,31,32,33,34,35,37,38,39,40,41,42,43,44,45,46,47,48,49,50,51,52,53,54,55,56,57]. This risk was primarily attributed to inappropriate exclusion criteria, including the failure to explicitly exclude women presenting with clinical chorioamnionitis, as well as the exclusion of participants due to incomplete data.

Additionally, studies in which the cut-offs were not established a priori based on external evidence or consensus but derived from the study data were considered to have a high risk of bias. Regrettably, all of the plasma studies and an accountable proportion of AF and CVF studies did not use predefined cut-offs [11,12,13,18,20,22,23,25,27,28,30,31,32,33,34,36,41,42,44,45,48,49,50,51,52,53,54,55,56,57].

Only 12 studies [12,26,27,33,37,39,41,42,43,44,46,55] reported that placental assessments were blinded. This underreporting may be due to the fact that pathologists are usually unaware of the pregnant women’s lab results, and blinding is subsequently implied. However, a strict approach was adopted, categorizing studies that did not explicitly mention blinding as biased. Studies with poorly defined HCA reference standards were also considered biased [47,51,53]. 

Regarding the last domain, flow and timing, a ≤120 h interval between sampling and delivery was deemed suitable in order for the correlation between the index test and the outcome at placental evaluation to be maintained. The majority of studies measured IL-6 only upon admission and did not repeat the test closer to birth, leaving only 14 studies with a low risk of bias [12,25,29,31,34,37,38,39,40,41,48,50,52,55].

The majority of studies were judged to be at a high risk of bias (26/43), while a substantial proportion raised concerns (14/46). Only three studies were assessed as low risk (two in CVF and one in plasma), precluding the possibility of conducting a meaningful sensitivity analysis. Performing a sensitivity analysis including only those studies or even studies with “some concerns”, still exhibited considerable methodological limitations, meaning that such an analysis would not have appreciably reduced bias or improved the validity of the pooled estimates. Furthermore, we compared the predefined and optimal AF cut-offs to assess potential differences in diagnostic performance, without observing statistical significance (Table 7). Similarly, no other tested covariates—including publication year, assay type, time to delivery <120 h—were found to be significant (*p* < 0.05).

For CVF and plasma a similar approach was even more restricted by the number of available studies, rendering subgroup analyses statistically underpowered and interpretively fragile. For plasma, despite testing multiple study-level characteristics, the analysis did not identify any statistically significant sources of this variation. Regarding CVF, the analysis revealed a key factor influencing the results: studies defining imminent delivery within a <120 h window reported a significantly higher diagnostic accuracy (*p* = 0.0107). Substantial residual heterogeneity remains, indicating that other unmeasured factors are likely to contribute to the observed variation.

## 4. Discussion

In this systematic review, we analyzed the diagnostic performance of IL-6 extracted from three different body fluids—blood, AF, and CVF—to identify patients with HCA. We included forty-three studies. We found that IL-6’s overall performance was very good for AF and CVF with AUCs of 0.82 and 0.85, respectively, and plasma’s overall performance was also good with an AUC of 0.79. According to our findings, CVF IL-6 could serve as a point-of-care (POC) test to stratify risk in women with PPROM for the outcome of HCA, having an excellent sensitivity and a great diagnostic performance (AUC 0.91) for this subgroup.

**Figure 9:**
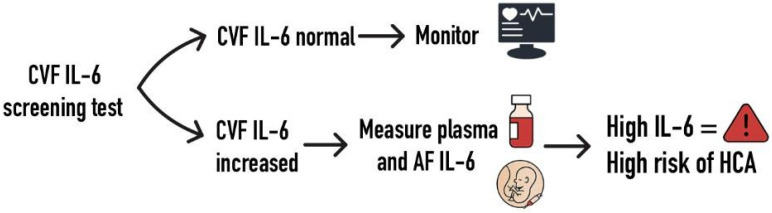
Proposed diagnostic algorithm.

For patients with an increased POC CVF IL-6 count, an additional plasma and AF measurement would add diagnostic value for suspected HCA. The PTL subgroup could benefit from AF IL-6 measurement. Thus, a protocol of POC CVF IL-6 measurement, followed by AF and plasma sampling in high-risk patients, could assist clinicians in therapeutic and further diagnostic decisions. However, these findings represent preliminary evidence requiring rigorous validation before clinical adoption. This framework remains hypothetical without prospective trials confirming its impact on outcomes (Figure 9).

Plasma IL-6 was measured in patients with PPROM and PTL using an optimal cut-off that was consistent across studies discussing those two sub-populations. This data explains the consistency of diagnostic accuracy between those two subgroups. In addition, studies that examined AF IL-6 in PTL patients usually had predefined cut-offs compared with the majority of studies that examined AF IL-6 in PPROM patients, which had either predefined or optimal cut-offs. For this reason, despite the similarity in specificity, sensitivity was higher in the PTL AF IL-6 group, reflecting a higher false-negative rate due to adjusted cut-offs. Standardization of IL-6 measurement protocols and threshold values emerges as a critical consideration for clinical implementation as inconsistent thresholds could lead to misclassification, increasing diagnostic heterogeneity.

We conducted a literature review and tracked two meta-analyses that examined the diagnostic value of IL-6, but in a mixed population of HCA and/or funisitis and only in plasma and AF [58,59].

Etyang et al. meta-analyzed plasma IL-6, among other plasma biomarkers, to assess its diagnostic performance for HCA or funisitis [58]. According to their high-sensitivity ROC analysis, which included five studies, plasma IL-6 demonstrated a very good diagnostic performance with a sensitivity of 0.52 and a specificity of 0.82, without providing AU-ROC values in their study. CRP and PCT had similar diagnostic performance and a combination of those biomarkers in future RCTs would be of great interest [58].

Lanzarone et al. also meta-analyzed plasma and AF IL-6 with the same outcomes as Etyang et al. [59]. Among five studies that analyzed plasma IL-6 for the diagnosis of HCA or funisitis, the pooled sensitivity was 59.6% (95% CI: 51.0–67.7%) and the pooled specificity was 82.6% (95% CI: 75.4–88.4%). Among four studies that analyzed AF IL-6, the pooled sensitivity was 58.5% (95% CI 51.7–65.0%) and the pooled specificity was 80.4% (95% CI: 73.2–86.4%). Both meta-analyses mention high heterogeneity among their respective populations. Among other biomarkers, including plasma WBCs, CRP, and PCT and AF WBCs, plasma and AF IL-6 had the second and third best diagnostic ORs (10.1 and 6.19, respectively), lacking only from AF WBCs’ diagnostic performance (OR 15.25).

While acknowledging that other traditional inflammatory biomarkers have shown promising potential and numerous novel biomarkers for HCA are currently under exploration, our systematic review deliberately focused on IL-6. Since there is no ideal biomarker for any condition, this strategic focus was motivated by a critical advancement in its clinical applicability: the development of POC IL-6 testing. Traditional laboratory-based methods for biomarker measurement necessitate specialized infrastructure and involve significant processing time, inherently limiting their utility in many clinical settings, particularly those requiring rapid decision-making or operating with resource constraints. In contrast, the emergence of POC IL-6 testing represents a transformative advancement in clinical utility.

The clinical implications of early HCA detection, across diverse clinical environments, including those in which invasive procedures or complex laboratory support may be limited, extend beyond diagnosis to influence multiple aspects of perinatal care. For patients identified as low risk through a preliminary assessment, a conservative monitoring approach could enable significant reductions in unnecessary resource utilization. Conversely, early identification of high-risk cases facilitates prompt obstetric consultation and timely escalation of care and close monitoring, reducing the likelihood of emergency procedures and improving coordination of maternal–fetal interventions. Overall, routine pre-birth assessment for HCA can shift care toward higher-value, evidence-driven pathways that better align resources with patient risk.

The strengths of our study include the meta-analysis of CVF IL-6 for the diagnosis of HCA, which is a less invasive, point-of-care procedure with high sensitivity that had not been previously meta-analyzed. Using a broad search strategy, we were able to include a large number of important articles that had not been previously analyzed in any other research on this topic. By focusing on IL-6 and deriving data from studies that measured it in several body fluids, such as plasma, AF, and CVF, and in discernible subgroups, such as PPROM and PTL patients, we were able to compare diagnostic performances and indicate a possible diagnostic pathway for further investigation and real-world validation.

The limitations of our study include the inability to include studies that did not provide 2 × 2 data despite corresponding with their respective authors. Substantial heterogeneity is evident in the sROC scatter plot. While variables such as assay method and time to delivery are biologically and clinically plausible sources of variation, they did not emerge as statistically significant modifiers in this analysis. This likely reflects the limited number of included studies, small sample sizes, and high risk of bias, all of which constrain statistical power and obscure true sources of heterogeneity. The absence of detectable associations should therefore be interpreted as a limitation of the evidence base rather than as a negation of underlying effects. Unmeasured confounders and context-specific interactions may further contribute to the observed dispersion but cannot be reliably explored given the current data. The lack of evidence in the CVF PTL group creates a gap in the literature regarding the diagnostic value of IL-6. The strategic decision to focus on IL-6, despite providing valuable insights into its diagnostic behavior, leaves room for further investigation compared with other biomarkers. The high risk-of-bias detection reflects the nature of the included studies, which were mostly retrospective, non-randomized, and lacking homogenous protocols in patient selection and measurement methods. We chose not to use the latest definition of “triple I” regarding HCA infection due to a lack in the literature referring to this emerging approach, setting a pivot for investigation for the applicability of our findings on this definition [60]. Potential limitations also include language bias, as non-English studies may have been underrepresented, also given the chosen databases that were screened. The registration of our protocol to INPLASY afterwards and not to the PROSPERO before is also a limitation.

While IL-6 shows strong diagnostic potential, its standalone utility has to be evaluated in comparison or even more in combination with other commercially available biomarkers in POC settings. Multi-marker panels including CVF IL-6, MMP-8, and IL-8 have been studied in this setting for HCA prediction with acceptable results for each biomarker alone (AUCs 0.61–0.77). These data could significantly enhance diagnostic precision by addressing the heterogeneity of host responses [46]. As demonstrated in a recent external validation study, a panel combining vaginal IL-6 with alpha-fetoprotein achieved excellent performance, particularly a high negative predictive value (NPV) of 92.9% for ruling out inflammation [61]. Yet, no large-scale studies have validated such combinations for HCA. This underscores a critical direction for future research: the development and, crucially, the prospective external validation of multi-marker, non-invasive panels. Machine learning algorithms could address the heterogeneity in biomarker performance by developing context-specific diagnostic models that dynamically adjust cut-off values based on patient characteristics such as gestational age, presence of PPROM or PTL, and other clinical parameters [62]. These AI-driven approaches could integrate multiple biomarkers with clinical data to create sophisticated prediction models that outperform single-marker strategies. The ultimate goal should be to move beyond hypothetical frameworks, such as the one proposed in our review, toward large-scale, multi-center prospective trials. Such trials are essential to determine whether the implementation of a non-invasive, risk-stratification tool—whether based on IL-6 alone or a multi-marker panel—translates into tangible improvements in clinical management, antibiotic stewardship, and, most importantly, maternal and neonatal outcomes. 

## 5. Conclusions

These findings suggest that CVF IL-6 could serve as a valid, non-invasive screening test for pregnant women to stratify risk for HCA, while a combination of AF, CVF, and plasma IL-6 could potentially serve as diagnostic tool for clinicians on this purpose. They also emphasize the urgent need for future primary studies designed with enhanced methodological robustness and standardized protocols to elucidate the true sources of heterogeneity and strengthen the evidence foundation.

## Figures and Tables

**Table 1:** Study characteristics.

Author	Year	Study Design	Fluid	N	Cut-off	Subgroup	Gestational Age	Assay Method	Sampling Timing
Joo [11]	2021	Retro	AF	150	2.60 (pre)	PPROM	24 + 0 to 33 + 6	ELISA	Admission
Fulova [18]	2021	Pros	AF	62	1.00 (pre)	PPROM	22 + 0 to 34 + 0	POC	Admission
Lee [19]	2018	Retro	AF	74	1.48 (opt)	PPROM	21 + 0 to 34 + 0	Luminex	Admission
Musilova [20]	2018	Pros	AF	159	0.74 (pre)	PPROM	34 + 0 to 36 + 6	POC	Admission
Lee [21]	2017	Retro	AF	151	1.5 (opt)	PPROM	21 + 0–33 + 6	ELISA	Admission
Musilova [22]	2017	Retro	AF	154	0.74 (pre)	PPROM	24 + 0 to 36 + 6	POC	Admission
Musilova [23]	2016	Pros	AF	168	0.74 (pre)	PPROM	24 + 0 to 36 + 6	POC	Admission
Kim [24]	2016	Retro	AF	146	2.40 (opt)	PPROM	20 + 0 to 33 + 6	ELISA	Admission
Chaemsaithong [25]	2016	Retro	AF	26	0.74 (pre)	PPROM	20 + 0 to 35 + 0	POC	-
Kacerovsky [26]	2014	Pros	AF	124	0.32 (opt)	PPROM	24 + 0 to 36 + 6	POC	-
Cobo [27]	2012	Pros	AF	107	0.02 (pre)	PPROM	23 + 0 to 36 + 6	Luminex M	Admission
Cobo [28]	2011	Pros	AF	63	1.43 (pre)	PPROM	<34 + 0	ELISA	First 48 h
Lee [29]	2022	Retro	AF	100	5.57 (opt)	PPROM	24 + 0 to 34 + 0	ELISA	Admission
Leaños-Miranda [30]	2021	Pros	AF	452	2.60 (pre)	PTL	<36 + 0	ELISA	-
Chaemsaithong [31]	2016	Retro	AF	54	2.60 (pre)	PTL	20 + 0 to 35 + 0	ELISA	-
Romero [32]	2015	Retro	AF	125	2.60 (pre)	PTL	20 + 0 to 36 + 6	ELISA	-
Combs [33]	2014	Pros	AF	218	2.60 (pre)	PTL	15 + 0 to 36 + 6	ELISA	Admission
Negishi [34]	1996	Pros	AF	18	0.60 (pre)	PTL	24 + 0 to 36 + 0	ELISA	-
Tsuda [35]	1998	Pros	AF	110	3.5 (opt)	PTL	16 + 0 to 32 + 0	ELISA	Admission
Hillier [36]	1993	Pros	AF	26	1.5 (pre)	PTL	24 + 0 to 34 + 0	ELISA	7 days before
Oh [37]	2011	Retro	AF	99	2.60 (opt)	PPROM/PTL	21 + 0 to 35 + 0	ELISA	Admission
Miura [38]	2011	Retro	AF	56	11.27 (opt)	TA PTL/PPROM	24 + 0 to 35 + 0	ELISA	Admission
Yoon [39]	1995	Pros	AF	29	17 (opt)	PPROM/PTL	24 + 0 to 35 + 0	ELISA	Admission
El-Ghazaly [12]	2022	Pros	CVF	110	0.1 (pre)	PPROM	24 + 0 to 34 + 0	POC	Admission + TOP/induction
Balciuniene [40]	2021	Pros	CVF	156	1.39 (opt)	PPROM	22 + 0 to 34 + 6	ELISA	Every second day
Eleje [41]	2020	Pros	CVF	48	0.1 (pre)	PPROM	24 + 0 to 36 + 6	POC	Before DCE
Kayem [42]	2017	Pros	CVF	141	0.1 (pre)	PPROM	24 + 0 to 37 + 0	POC	7 days before
Lucovnik [43]	2011	Pros	CVF	42	1.045 (opt)	PPROM	23 + 6 to 31 + 6	CLIA	Admission
Kayem [44]	2005	Pros	CVF	51	0.1(pre)	PPROM	24 + 6 to 34 + 6	POC	Admission
Abdelazim [45]	2013	Pros	CVF	120	0.1 (pre)	PPROM	34 + 0 to 37 + 0	POC	-
Park [46]	2023	Retro	CVF	134	0.096 (opt)	PTL	23 + 0 to 34 + 0	ELISA	Admission
Lockwood [47]	1994	Pros	CVF	12	0.25 (opt)	PPROM/PTL	24 + 0 to 36 + 0	ELISA	<28 days before
Kong [13]	2023	Retro	PL	206	8.252 (opt)	PPROM	30 + 0 to 35 + 0	CLIA	Within 12 h
Park [48]	2019	Retro	PL	82	3.87 (opt)	PPROM	23 + 0 to 34 + 6	ELISA	Admission
Martinez-Portilla [49]	2019	Pros	PL	47	19.5 (opt)	PPROM	26 + 0 to 36 + 6	ELISA	Admission
Sayed Ahmed [50]	2016	Pros	PL	60	8.5 (opt)	PPROM	24 + 0 to 34 + 0	ELISA	Admission + TOP/onset of labor
Gulati [51]	2012		PL	45	8 (opt)	PPROM	24 + 0 to 34 + 0	ELISA	Admission + Onset of labor + 48 h + day 7
Park [52]	2019	Retro	PL	74	5.88 (opt)	PTL	23 + 0 to 34 + 6	ELISA	Admission
Torbé [53]	2007	Pros	PL	48	40 (opt)	PTL	24 + 0 to 34 + 0	ELISA	-
Koyama [54]	2000	Pros	PL	38	7.5 (opt)	PTL	23 + 0 to 36 + 0	ELISA	-
Maeda [55]	1997	Pros	PL	29	7.5 (opt)	PTL	22 + 0 to 34 + 0	ELISA	Delivery
Greig [56]	1997	Pros	PL	20	6 (opt)	PTL–PTD	22 + 0 to 34 + 0	ELISA	-
Skrablin [57]	2007	Pros	PL	47	29.1 (opt)	PTL/PPROM	27 + 0 to 33 + 0	ELISA	Admission

Abbreviations: AF: amniotic fluid, CVF: cervicovaginal fluid, Retro: retrospective, Pros: prospective, opt: optimal, pre: prespecified, ELISA: enzyme-linked immunosorbent assay, POC: point of care (test), PTD: preterm delivery.

**Table 2:** AF—diagnostic accuracy parameters in overall analysis.

Measure	AF Overall (95% CI)
DOR	9.53 (6.59–13.77)
LR+	3.90 (2.83–5.37)
LR−	0.41 (0.32–0.52)
AUC	0.82 (0.78–0.85)

**Table 3:** Plasma—diagnostic accuracy parameters in overall analysis.

Measure	Plasma Overall (95% CI)
DOR	9.06 (4.88–16.82)
LR+	3.30 (2.52–4.31)
LR−	0.36 (0.24–0.55)
AUC	0.79 (0.76–0.83)

**Table 4:** CVF—diagnostic accuracy parameters in overall analysis and in the sensitivity analysis of PPROM.

Measure	CVF Overall (95% CI)	CVF PPROM (95% CI)
DOR	16.16 (3.13–83.37)	33.03 (5.56–196.24)
LR+	3.51 (1.85–6.68)	4.62 (2.30–9.30)
LR−	0.21 (0.08–0.60)	0.14 (0.05–0.43)
AUC	0.85 (0.82–0.88)	0.91 (0.88–0.93)

**Table 5:** AF—subgroup analysis.

Measure	AF PPROM (95% CI)	AF PTL (95% CI)	z	*p*
DOR	5.87 (4.49–7.66)	20.80 (7.93–54.58)	2.479	0.013
LR+	3.25 (2.52–4.19)	4.91 (1.79–13.44)	0.776	0.437
LR−	0.55 (0.47–0.65)	0.24 (0.13–0.44)	2.591	0.010
AUC	0.76 (0.72–0.80)	0.88 (0.85–0.91)	5.004	<0.00001

**Table 6:** Plasma—subgroup analyses.

Measure	Plasma PPROM (95% CI)	Plasma PTL (95% CI)	z	*p*
DOR	5.82 (3.16–10.72)	9.93 (3.58–27.56)	0.881	0.379
LR+	2.89 (2.09–3.99)	3.12 (2.05–4.74)	0.284	0.776
LR−	0.50 (0.35–0.69)	0.31 (0.15–0.64)	1.128	0.259
AUC	0.80 (0.76–0.83)	0.77 (0.74–0.81)	0.860	0.390

**Table 7:** AF—cut-off analyses.

Measure	Predefined (95% CI)	Optimal (95% CI)	z	*p*
DOR	8.90 (5.36–14.76)	9.93 (5.89–16.76)	1.112	0.266
LR+	4.06 (2.49–6.62)	3.51 (2.55–4.84)	0.483	0.629
LR−	0.46 (0.34–0.62)	0.35 (0.25–0.49)	0.297	0.766
AUC	0.80 (0.76–0.83)	0.83 (0.79–0.86)	1.183	0.237

## Data Availability

The raw data supporting the conclusions of this article will be made available by the authors on request.

## Supplementary Materials

The following supporting information can be downloaded at: https://www.mdpi.com/article/10.3390/biomedicines13112577/s1. Table S1: Model-fitting and heterogeneity statistics for the bivariate meta-analysis model of sensitivity and specificity.
