# Supplementary material for: Interleukin 6 for the Prediction of Chorioamnionitis: A Systematic Review and Meta-Analysis
Source: Biomedicines. 2025 Oct 22;13(11):2577. doi: 10.3390/biomedicines13112577 (PMC12650506; doi:10.3390/biomedicines13112577)
Supplement: Supplementary file 1 [file biomedicines-13-02577-s001.zip › biomedicines-3919600-supplementary.pdf]

Supplementary Table S1

Model-fitting and heterogeneity statistics for the bivariate meta-analysis model of sensitivity and specificity.

Analyses	Tau-squared*	I^2§	rho†	Likelihood ratio test, random-effects versus fixed-effects model‡
**Analysis on AF**				
Overall	0.37; 0.91; 1.20	68.86%; 82.16%; 75.58%	-0.81	305.97; df=3; p<0.0001
PPROM	0.00; 0.48; 0.55	0.01%; 81.99%; 71.43%	-1.00	97.30; df=3; p<0.0001
PTL	0.79; 1.14; 2.97	57.64%; 71.44%; 75.93%	-0.87	185.64; df=3; p<0.0001
Mixed PPROM-PTL	0.00; 0.63; 0.42	0.01%; 64.82%; 57.71%	-1.00	8.79; df=3; p<0.032
**Analysis on CVF**				
Overall	0.00; 2.25; 0.75	0.08%; 67.91%; 72.95%	1.00[a]	90.85; df=3; p<0.0001
PPROM	0.08; 1.93; 0.76	40.74%; 77.17%; 73.13%	0.97[a]	64.47; df=3; p<0.0001
**Analysis on plasma**				
Overall	0.02; 0.79; 0.02	29.92%; 73.86%; 6.69%	-0.32	26.76; df=3; p<0.0001
PPROM	0.00; 0.26; 0.01	0.01%; 66.09%; 3.68%	-1.00	6.37; df=3; p = 0.095
PTL	0.00; 0.79; 0.01	0,05%; 69.23%; 2.14%	-1.00	6.13; df=3; p=0.106

* Generalized; sensitivity; specificity.

§ Generalized; sensitivity; specificity.

† Correlation between sensitivity and specificity on the logit scale.

‡ Chi-squared statistic; degrees of freedom (df); p-value.

[a] There was a study with 100% sensitivity, hence, the positive rho value.
